# ACSL1 Regulates TNFα-Induced GM-CSF Production by Breast Cancer MDA-MB-231 Cells

**DOI:** 10.3390/biom9100555

**Published:** 2019-10-01

**Authors:** Reeby Thomas, Fatema Al-Rashed, Nadeem Akhter, Fahd Al-Mulla, Rasheed Ahmad

**Affiliations:** 1 Microbiology & Immunology Department, Dasman Diabetes Institute, Kuwait City, Kuwait; Reeby.Thomas@dasmaninstitute.org (R.T.); fatema.alrashed@dasmaninstitute.org (F.A.-R.); nadeem.akhter@dasmaninstitute.org (N.A.); 2 Genetics and Bioinformatics Department, Dasman Diabetes Institute, Kuwait City, Kuwait; fahd.almulla@dasmaninstitute.org

**Keywords:** ACSL1, TNFα, GM-CSF, MDA-MB-231

## Abstract

Overexpression of granulocyte-macrophage colony-stimulating factor (GM-CSF) in different types of cancer is associated with tumor growth and progression. Tumor necrosis factor-α (TNFα) is involved in the induction of GM-CSF in different cells; however, the underlying molecular mechanism in this production of GM-CSF has not been fully revealed. Recently, it was noted that TNFα mediates inflammatory responses through long-chain acyl-CoA synthetase 1 (ACSL1). Therefore, we investigated the role of ACSL1 in the TNFα mediated production of GM-CSF. Our results showed that MDA-MB-231 cells displayed increased GM-CSF mRNA expression and secretion after incubation with TNFα. Blocking of ACSL1 activity in the cells with triacsin C markedly suppressed the secretion of GM-CSF. However, inhibition of β-oxidation and ceramide biosynthesis were not required for GM-CSF production. By small interfering RNA mediated knockdown, we further demonstrated that TNFα induced GM-CSF production was significantly diminished in ACSL1 deficient cells. TNFα mediated GM-CSF expression was significantly reduced by inhibition of p38 MAPK, ERK1/2 and NF-κB signaling pathways. TNFα induced phosphorylation of p38, ERK1/2, and NF-κB was observed during the secretion of GM-CSF. On the other hand, inhibition of ACSL1 activity attenuates TNFα mediated phosphorylation of p38 MAPK, ERK1/2, and NF-κB in the cells. Importantly, our findings suggest that ACSL1 plays an important role in the regulation of GM-CSF induced by TNFα in MDA-MB-231 cells. Therefore, ACSL1 may be considered as a potential novel therapeutic target for tumor growth.

## 1. Introduction

Granulocyte-macrophage colony-stimulating factor (GM-CSF), also known as colony-stimulating factor 2 (CSF2), is a growth factor/cytokine which regulates the maturation and differentiation of hematopoietic cells [1]. Dysregulation of GM-CSF is involved in chronic inflammatory diseases by the migration of myeloid cells to inflammation sites, sustaining the survival of target cells and promoting the renewal of macrophages [2]. In this context, a pathogenic role of elevated GM-CSF has been reported in autoimmune diseases that are dependent on cellular immune responses, such as multiple sclerosis (MS) and rheumatoid arthritis (RA) [3,4]. A protective role of GM-CSF has also been reported in some autoimmune diseases such as myasthenia gravis (MG), Hashimoto’s thyroiditis (HT), inflammatory bowel disease (IBD), and systemic lupus erythematosus (SLE) [5,6,7,8]. Many studies show the involvement of GM-CSF in promoting tumor growth and progression. GM-CSF promotes cancer cell proliferation and migration in a wide variety of tumors and multiple cancer models. In this connection, multiple cancer models show constitutive GM-CSF protein expression and secretion, often together with its conjugate receptors [9]. In colorectal cancer patients, an increased level of GM-CSF in serum is considered as a potential diagnostic and prognostic marker for poor prognosis [10]. Furthermore, high GM-CSF protein levels have been related to tumor growth and poor prognosis in patients with head/neck and lung cancers [11].

GM-CSF is produced by multiple cell types such as activated monocytes, macrophages, T cells, B cells, fibroblasts, mast cells, vascular endothelial cells, and a wide variety of cancer cell types [8]. GM-CSF expression is induced by lipopolysaccharide and proinflammatory cytokines, including IL-1 [12], IL-6 [13], and tumor necrosis factor-α (TNFα) [14]. Increased circulating levels of TNFα and GM-CSF have been found in inflammatory disorders and multiples cancers [15]. However, the mechanism by which TNFα induces GM-CSF remains elusive. Herein, we determined whether TNFα could induce GM-CSF production in MDA-MB-231 breast cancer metastatic cells, and if so, which signal transduction pathway(s) were involved. Since acyl-CoA synthetase 1 (ACSL1) is involved in TNFα mediated immune responses [16], we investigated the role of ACSL1 in the TNFα mediated GM-CSF production. We found that pharmacologic and genetic inhibition of ACSL1 suppressed TNFα-induced GM-CSF expression in MDA-MBA-231 cells along with the activation of P38 MAPK, ERK1/2, and NF-κB. However, there is no role of β-oxidation and ceramide biosynthesis in TNFα induction of GM-CSF in MDA-MB-231. 

## 2. Results

### 2.1. TNFα Induces GM-CSF Gene Expression in Human MDA-MB-231 Cells 

**Figure 1:**
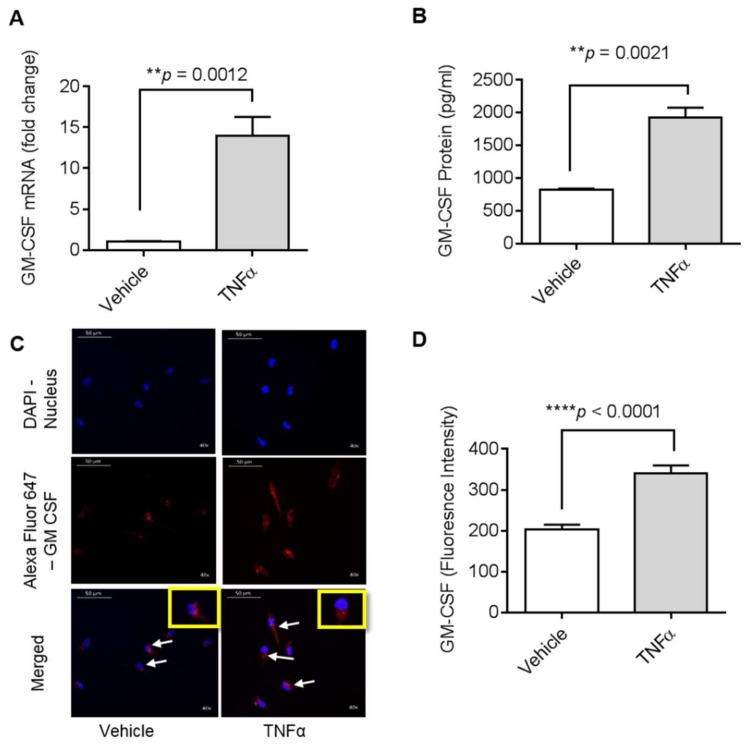
Effect of tumor necrosis factor-α (TNFα) on granulocyte-macrophage colony-stimulating factor (GM-CSF) production in human MDA-MB-231 cells. MDA-MB-231 cells were cultured in 6-well plates at a concentration of 1 × 10^6^ cells/well. Cells were treated with vehicle and TNFα (2 ng/mL), separately. After 24 h incubation, cells and supernatants were collected. (**A**) Total cellular RNA was isolated and GM-CSF mRNA expression was determined by real-time PCR. (**B**) Secreted GM-CSF in culture media was determined by ELISA. (**C**) MDA-MB-231 cells were treated with vehicle or TNFα for 24 h and then were stained with GM-CSF (red) and DAPI (blue). White arrows indicate typical stained cells. (**D**) GM-CSF fluorescence intensity is shown. The results obtained from three independent experiments are shown. All data are expressed as mean ± SEM (*n* ≥ 3). ** *p* < 0.01, **** *p* < 0.0001 versus vehicle.

Previous studies have shown that TNFα activates MMP-9 gene expression in MDA-MB-231 cells [17]. However, since GM-CSF induction by TNFα in MDA-MB-231 cells has not yet been studied, we looked at the impact of TNFα on the regulation of GM-CSF gene expression in these cells. To examine whether TNFα can induce gene expression of GM-CSF, we treated MDA-MB-231 cells with TNFα for 24 h in serum-free media. Our data show (Figure 1A) that GM-CSF mRNA expression levels were significantly higher (14.02-fold; *p* < 0.001) in TNFα-treated MDA-MB-231 cells than those of controls (cells treated with vehicle only). Concordantly, GM-CSF protein levels (Figure 1B) were significantly higher in MDA-MB-231 cells supernatant after stimulation with TNFα (1820.67 pg/mL; *p* < 0.0001). Confocal microscopy along with fluorescence intensity of MDA-MB-231 cells also showed that there was a significant increase in the expression of GM-CSF in the cells treated with TNFα (Figure 1C,D).

### 2.2. TNFα Induced GM-CSF Production is Suppressed by Inhibition of ACSL1 

**Figure 2:**
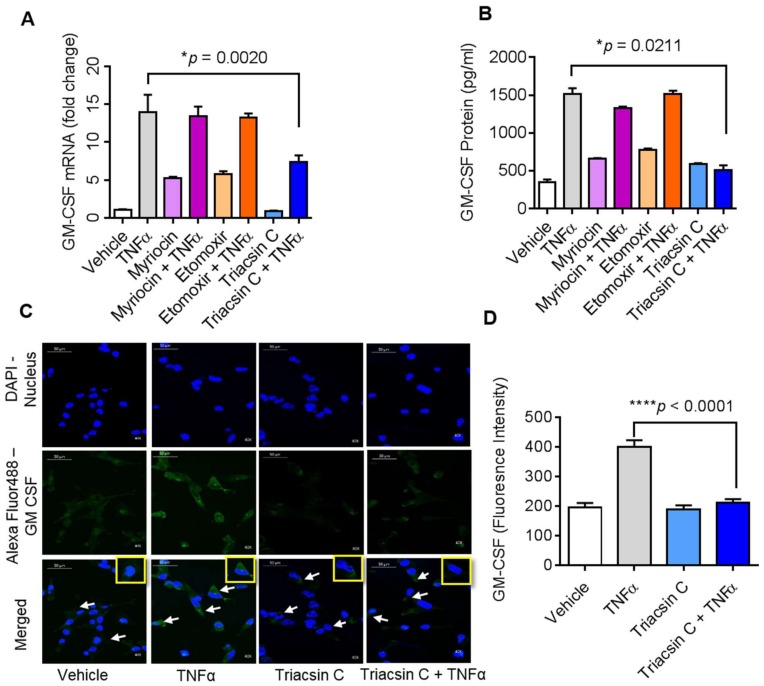
Effect of acyl-CoA synthetase 1 (ACSL1) inhibition on GM-CSF production in MDA-MB-231 cells. MDA-MB-231 cells were pretreated with a long-chain ACSL1 inhibitor (triacsin C, 5 μM), a serine palmitoyltransferase inhibitor (SPT-1) involved in sphingolipid biosynthesis (myriocin, 1 μM), a carnitine palmitoyltransferase 1 (CPT-1) inhibitor (etomoxir, 10 μM), or vehicle for 1 h and then incubated with TNFα for 24 h. (**A**) GM-CSF mRNA was determined by real-time PCR. (**B**) Secreted GM-CSF in culture media was determined by ELISA. (**C**) MDA-MB-231 cells were stained with GM-CSF (green) and DAPI (blue). White arrows indicate typical stained cells. (**D**) GM-CSF fluorescence intensity was determined. All data are expressed as mean ± SEM (*n* ≥ 3). * *p* < 0.05, **** *p* < 0.001 versus vehicle.

Emerging evidence suggests that ACSL1 is involved in TNFα mediated immune regulation [16,18]. We therefore examined whether ACSL1 was required for TNFα induced GM-CSF production by MDA-MB-231 cells, and we used triacsin C to inhibit ACSL1 activity in MDA-MB-231 cells and measured the GM-CSF. Our data show that pretreatment of the MDA-MB-231 cells with triacsin C, followed by the exposure to TNFα, caused a significant inhibition in the expression of GM-CSF (Figure 2A,B; *p* < 0.05). Since TNFα activates GM-CSF gene expression via ACSL1 which directs fatty acids towards β-oxidation [19] and ceramide production [20], we asked whether these components play a role in TNFα induced GM-CSF production. To this end, MDA-MB-231 cells were treated with inhibitors of fatty acid oxidation (etomoxir) or ceramide synthesis (myriocin) prior to incubation with TNFα. We found that etomoxir and myriocin did not block the TNFα induced production of GM-CSF (Figure 2A,B).

Our confocal microscopy data along with fluorescence intensity of MDA-MB-231 cells also showed that there was a decrease in the expression of GM-CSF in triacsin C treated cells in response to TNFα (Figure 2C,D; *p* < 0.0001). Inhibition of ACSL1 significantly blocks the expression of GM-CSF in MDA-MB-231 cells.

### 2.3. ACSL1 Deficiency Suppresses TNFα-Induced GM-CSF 

**Figure 3:**
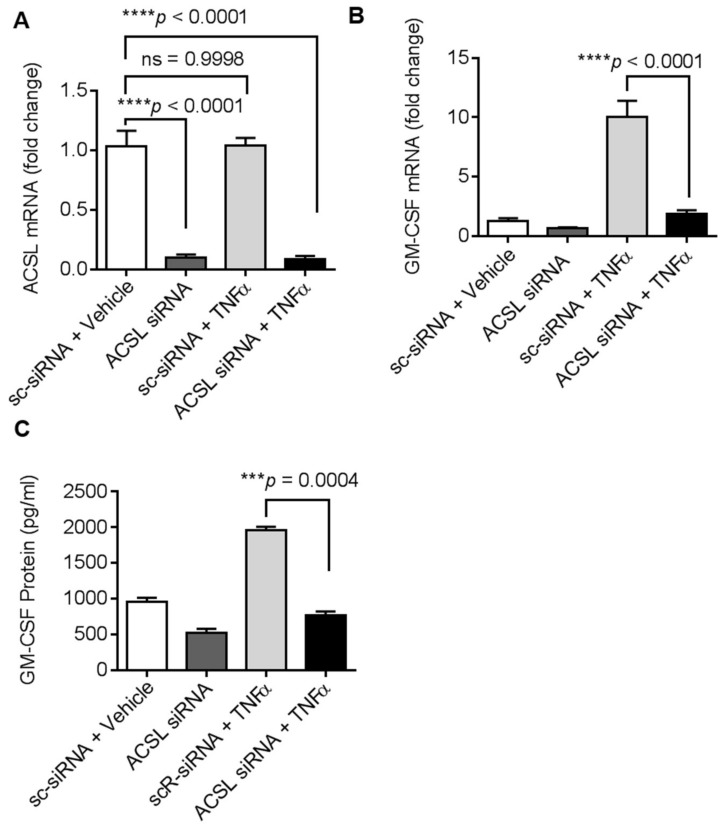
ACSL1 siRNA knockdown suppresses TNFα induced GM-CSF production. MDA-MB-231 cells were transfected with ACSL1 siRNA (targeting the human ACSL1 gene expression or scramble siRNA; a control siRNA). (**A**) After 36 h, real-time PCR was performed to measure ACSL1 expression to test knocking down efficiency. (**B**) ACSL1 deficient cells were then treated with vehicle and TNFα for 24 h. GM-CSF mRNA was determined by real-time PCR. (**C**) Secreted GM-CSF in culture media was determined by ELISA. All data are expressed as mean ± SEM (*n* ≥ 3). *** *p* < 0.001, **** *p* < 0.0001 versus vehicle.

To further verify if TNFα-induced GM-CSF in the MDA-MB-231 cells was dependent on ACSL1, we transfected cells with ACSL1 siRNA, which achieved more than 80% reduction in ACSL1 mRNA levels compared with scramble (control) siRNA (Figure 3A). As expected, the GM-CSF gene expression was significantly reduced in ACSL1 siRNA transfected cells after stimulation with TNFα as compared with scramble siRNA transfected cells (Figure 3B)**.** Similarly, GM-CSF protein expression was also significantly suppressed (*p* < 0.001) in ACSL1 deficient cells activated with TNFα (Figure 3C). Altogether, our results show that ACSL1 is a key effector in TNFα mediated GM-CSF production in MDA-MB-231 cells.

### 2.4. ACSL1 is involved in TNFα-Activated MAPK and NF-κB Signaling Pathways

**Figure 4:**
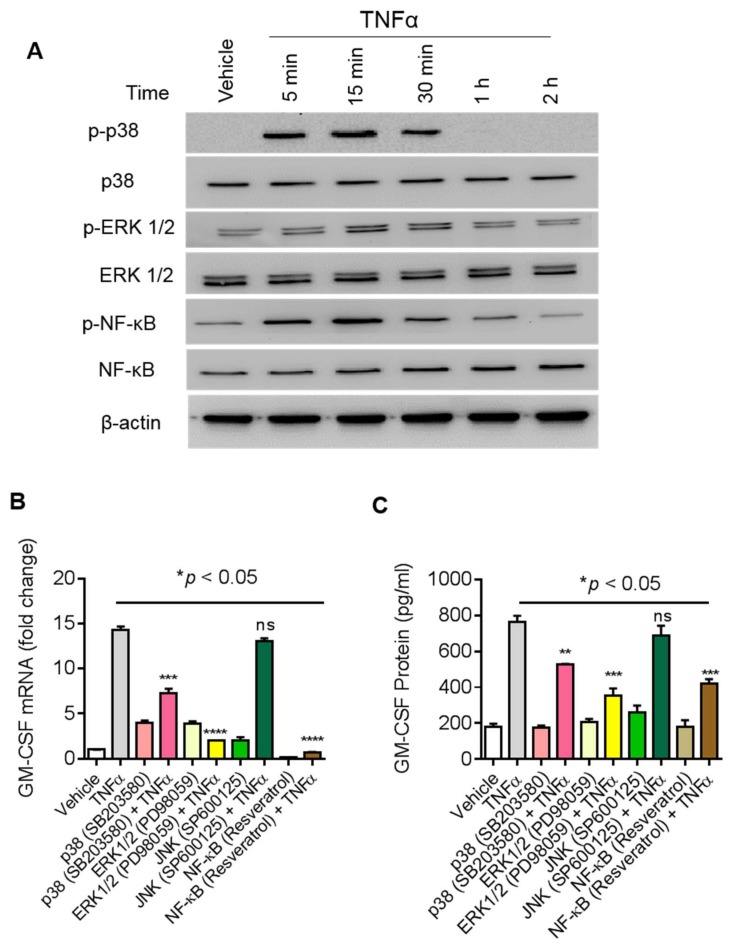
Effect of MAPK and NF-κB pathway inhibitors on TNFα induced GM-CSF production in MDA-MB-231 cells. (**A**) TNFα activates the MAPK/NF-κB signaling pathway. MDA-MB-231 cells were treated with TNFα for different time points and cell lysates were prepared as described in Materials and Methods. Samples were run on denaturing gels. Phosphorylated p38 MAPK, ERK1/2, and NF-κB are depicted in the upper panels and total respective proteins are shown in the lower panels. MDA-MB-231 cells were pretreated with p38 inhibitor (SB203580, 10uM; InvivoGen, San Diego, CA, USA) or ERK1/2 inhibitor (PD98059, 10 µM; InvivoGen, San Diego, CA, USA) or JNK inhibitor (SP600125, 10 µM) or NF-κB inhibitor (resveratrol, 1 µM) for 1 h and then treated with TNFα for 8 h. Cells and supernatants were collected. (**B**) Cells were used for the isolation of total RNA to assess the GM-CSF gene expression by real-time PCR. (**C**) Secreted levels of GM-CSF protein were determined in supernatants by ELISA. The results obtained from three independent experiments are shown. All data are expressed as mean ± SEM (*n* ≥ 3). **** *p* < 0.001 versus TNFα without respective inhibitors.

TNFα activates MAPK signaling (ERK1/2, p38, JNK) and NF-κB signaling pathways [21,22]. We next asked whether these molecules are also involved in the regulation of TNFα stimulated GM-CSF expression in MDA-MB-231 cells. First, we determined TNFα mediated phosphorylation of p38 MAPK, ERK1/2, and NF-κB for different time points (Figure 4A). TNFα treatment increases the phosphorylation of p38 MAPK, ERK1/2, and NF-κB in a time-dependent manner. Next, we found that TNFα mediated expression of GM-CSF mRNA was reduced (Figure 4B; *p* < 0.001) by treatment with inhibitors of either p38 MAPK (SB203580), ERK1/2 (PD98059), or NF-κB (resveratrol). Consistent with qRT-PCR results, GM-CSF levels in culture supernatants of MDA-MB-231 cells were significantly reduced (*p* < 0.001) after treatment with inhibitors of either MAPKs or NF-κB (Figure 4C). 

**Figure 5:**
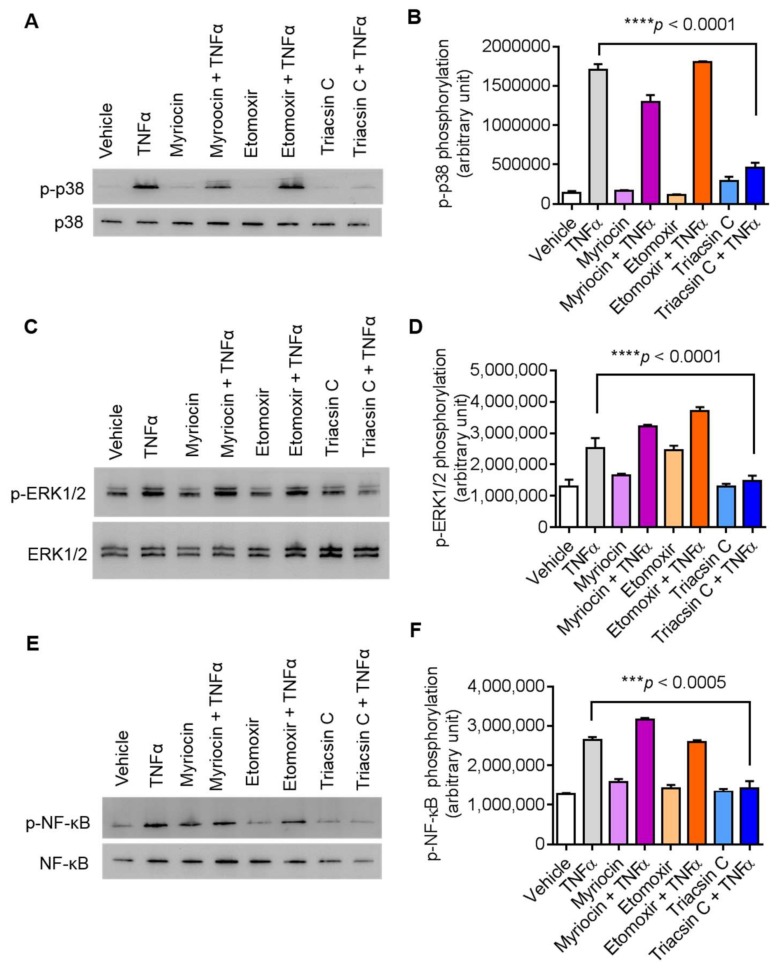
Inhibition of ACSL1 affects TNFα-activated MAPK and NF-κB signaling pathways in MDA-MB-231 cells. As shown in Figure 4A, TNFα treatment increases the phosphorylation of p38 MAPK, ERK1/2, and NF-κB in a time-dependent manner. MDA-MB-231 cells were pretreated with p38 inhibitor (SB203580, 10 μM), ERK1/2 inhibitor (PD98059, 10 μM), or NF-kB inhibitor (resveratrol, 1 μM) for 1 h and then treated with TNFα for 15 min. Cell lysates were prepared as described in Materials and Methods. Samples were run on denaturing gels. Immunoreactive bands were developed using an Amersham ECL Plus Western Blotting Detection System (GE Healthcare, Chicago, IL, USA) and visualized by Molecular Imager® VersaDoc^TM^ MP Imaging Systems (Bio-Rad Laboratories, Hercules, CA, USA). (**A**) Phosphorylated proteins p38 MAPK, (**C**) ERK1/2, and (**E**) NF-κB are depicted in the upper panels and total respective proteins are shown in the lower panels. (**B**, **D, F**) Phosphorylation intensity of p38 MAPK, ERK1/2, and NF-κB was quantified using Image Lab software (version 6.0.1, Bio-Rad, Hercules, CA, USA) and are presented in arbitrary units. All data are expressed as mean ± SEM (*n* ≥ 3). **** *p* < 0.001 versus TNFα without respective inhibitors.

In order to gain insight into ACSL1 function in TNFα induced activation of MAPK and NF-κB signaling proteins, we treated cells with inhibitors of ACSL1 prior to TNFα treatment. Our results showed that ACSL1 inhibition significantly reduced TNFα mediated phosphorylation of p38 MAPK, ERK1/2, and NF-κB (Figure 5A–F), indicating that p38 MAPK, ERK1/2, and NF-κB molecules were downstream of TNFα/ACSL1 signaling.

**Figure 6:**
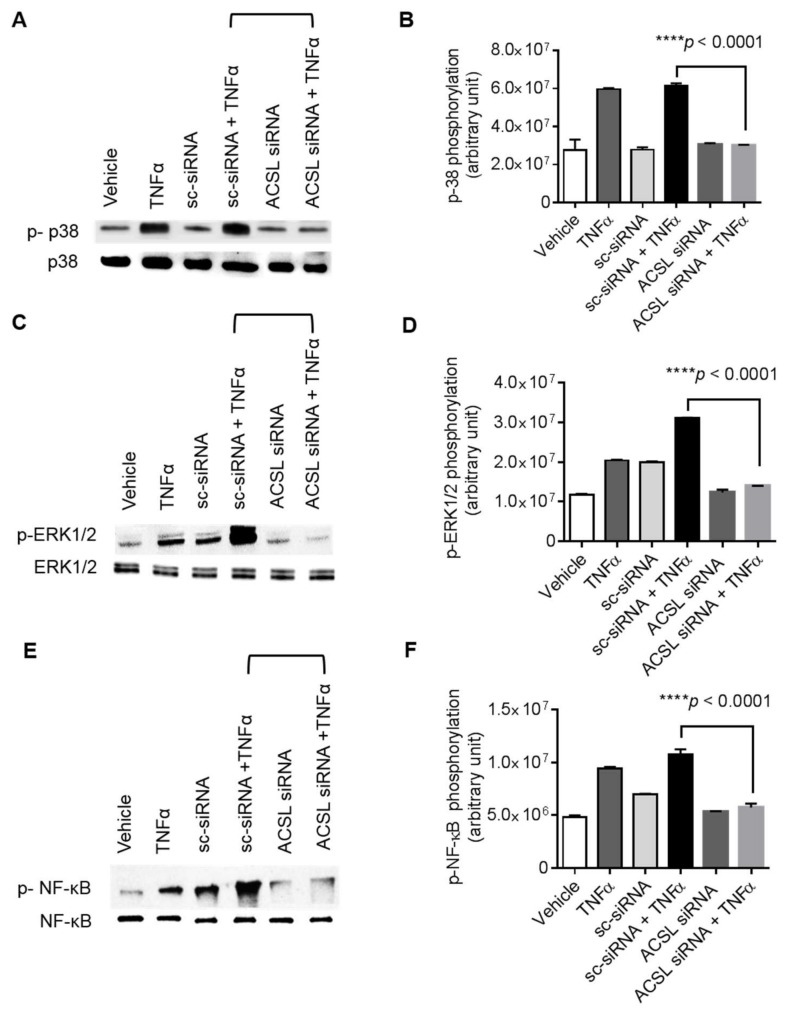
ACSL1 siRNA knockdown reduces the TNFα induced phosphorylation of p38 MAPK, ERK1/2, and NF-κB. MDA-MB-231 cells were transfected with ACSL1 siRNA (targeting the human ACSL1 gene expression) or scramble siRNA (a control siRNA) and incubated for 36 h. ACSL1 deficient cells were then treated with vehicle and TNFα for 15 min. Cell lysates were prepared as described in Materials and Methods. Samples were run on denaturing gels. Immunoreactive bands were developed using an Amersham ECL Plus Western Blotting Detection System (GE Healthcare, Chicago, IL, USA) and visualized by Molecular Imager® VersaDoc^TM^ MP Imaging Systems (Bio-Rad Laboratories, Hercules, CA, USA). (**A**) Phosphorylated proteins p38 MAPK, (**C**) ERK1/2, and (**E**) NF-κB are depicted in the upper panels and total respective proteins are shown in the lower panels. (**B**,**D**,**F**) Phosphorylation intensity of p38 MAPK, ERK1/2, and NF-κB was quantified using Image Lab Software (Bio-Rad) and presented in arbitrary units. All data are expressed as mean ± SEM. **** *p* < 0.001 versus TNFα-treated transfected cells with scramble siRNA.

To further verify the role of ACSL1 in TNFα induced activation of p38 MAPK, ERK1/2, and NF-κB signaling proteins, we transfected MDA-MB-231 cells with ACSL1 siRNA and scramble siRNA prior to TNFα treatment. Our results showed that ACSL1 silencing with siRNA significantly reduced TNFα-mediated phosphorylation of p38 MAPK, ERK1/2, and NF-κB (Figure 6A–F). Overall, our results support the conclusion that p38 MAPK, ERK1/2, and NF-κB molecules were downstream of TNFα/ACSL1 signaling.

## 3. Discussion

It is well established that overproduction of GM-CSF is involved in the pathogenesis of inflammatory diseases as well as tumor growth and progression [9,23]. In this regard, data indicated that breast cancer-derived GM-CSF supports local tumor growth and metastatic progression in preclinical models [24]. However, the underlying molecular mechanism in the overexpression of GM-CSF has not been fully understood. Our current findings demonstrate that TNFα induces GM-CSF in MDA-MB-231 breast cancer metastatic cells. Evidence from in vitro studies supports a role of TNFα in the regulation of GM-CSF in different cell lines [25]. Therefore, our results support the hypothesis that GM-CSF induction in MDA-MB-231 cancerous cells is regulated by TNFα. GM-CSF expression has been found, frequently together with the GM-CSF receptor, in a variety of tumor models, including small-cell lung carcinomas [26], meningiomas [27], skin carcinoma [28], gliomas [29], and head and neck squamous cell carcinomas (HNSCCs) [30]. It has been reported that high levels of IL-6 and TNFα are correlated with strong tumor invasiveness and poor prognosis of breast cancer [31], and our results show that TNFα induced GM-CSF in cancer cells, suggesting that TNFα may enhance tumor growth through the induction of GM-CSF.

Regarding molecular mechanism involved in TNFα stimulated induction of GM-CSF, recent studies have shown that ACSL1 deficiency prevents TNF𝛼 mediated induction of cell surface and secretory inflammatory markers in monocytic cells. Therefore, it was important to determine whether ACSL1 mediated signaling is involved in TNFα mediated regulation of GM-CSF gene expression. In this study, we presented two forms of evidence that TNFα induced production of GM-CSF depends on ACSL1. First, we observed that production of GM-CSF by TNFα is suppressed by pharmacologic inhibition of ACSL1 with triacsin C. Second, ACSL1 deficient MDA-MB-231 cells do not support the secretion of GM-CSF in response to stimulation with TNFα. This finding might also be explained by the observation described by Al-Rashed et al. showing that siRNA-mediated ACSL1 deficient monocytic cells demonstrate that TNFα induced inflammatory marker expression in monocytic cells requires ACSL1. Furthermore, ACSL1 deficiency prevents TNF𝛼 mediated induction of IL-1b and MCP-1 supporting the involvement of ACSL1 in the TNFα mediated production of cytokines and chemokines [16]. Since TNFα activates GM-CSF gene expression via ACSL1 which directs fatty acids towards β-oxidation [19] and ceramide production [20], MDA-Mb-231 cells were treated with inhibitors of fatty acid oxidation (etomoxir) or ceramide synthesis (myriocin) prior to incubation with TNFα. We found that etomoxir and myriocin did not block the TNFα induced production of GM-CSF. Our results suggest that ACSL1 mediates inflammatory effects without significant impact on β-oxidation and ceramide formation. In this connection, a study shows that ACSL1 mediates inflammatory effect without significant involvement of β-oxidation [32].

Next, we also looked at the ACSL1 function in TNFα induced activation of MAPK and NF-κB signaling proteins. It is well documented that TNFα stimulates MAPK and NF-κB signaling pathways involved in the regulation of several inflammatory cytokines that contribute to the pathogenesis of different inflammatory conditions [21,22]. It has been reported that TNFα induced GM-CSF secretion by human lung fibroblasts was partially blocked by ERK inhibitor and p38 MAPK inhibitor [33]. In this context, another study showed that p38 MAPK regulates TNFα induced RANTES and GM-CSF production in human bronchial epithelial cells [34]. Consistent with previous findings in different cells, we found that TNFα induced GM-CSF production by MDA-MB-231 cells was suppressed with the inhibition of p38 MAPK and ERK1/2, suggesting that different cell lines regulate the GM-CSF production in response to TNFα in a similar fashion, specifically in reference to p38 MAPK. Our data also show that TNFα induced phosphorylation of ERK1/2, p38 MAPK, and NF-κB in MDA-MB-231 cancer cells. Interestingly, our first results showed that blocking of ACSL1 activity inhibited the TNFα mediated phosphorylation of p38 MAPK, ERK1/2, and NF-κB in MDA-MB-231 cells. These results suggest that ACSL1 acts upstream of MAPK and NF-κB signaling pathways. 

In summary, the present study demonstrated that ACSL1 plays a crucial role in the regulation of TNFα mediated excessive production of GM-CSF associated with the inflammatory process that is involved in tumor growth. Therefore, targeting ACSL1 may be a new therapeutic strategy to prevent the inflammatory processes involved in cancer metastasis. Since TNFα can induce GM-CSF expression and secretion in several cancer cells, our results may furthermore suggest that a similar pattern of mechanisms may underlie TNFα induced production of GM-CSF by various cancer cells and tissues.

## 4. Materials and Methods

### 4.1. Cell Culture 

The MDA-MB-231 cell line is well established as a model for cancer research related to gene regulation. The MDA-MB-231 cell line is also being used for lipid research. Human MDA-MB-231 cells were purchased from American Type Culture Collection (ATCC, Manassas, VA, USA), and then grown in DMEM culture medium (Gibco, Thermo Fisher Scientific, Waltham, MA, USA) supplemented with 10% fetal bovine serum (Gibco, Thermo Fisher Scientific, Waltham, MA, USA), 2 mM glutamine (Gibco, Thermo Fisher Scientific, Waltham, MA, USA), 1 mM sodium pyruvate, 10 mM HEPES, 100 μg/mL Normocin, 50 U/mL penicillin and 50 μg/mL streptomycin (P/S; Gibco, Thermo Fisher Scientific, Waltham, MA, USA) and incubated at 37 °C (with humidity) in 5% CO_2_. 

### 4.2. Cell Stimulation

MDA-MB-231 cells were plated in 12-well plates (Costar, Corning Incorporated, Corning, NY, USA) at 1 × 10^6^ cells/well concentration unless otherwise indicated. Cells were treated with vehicle or TNFα (2 ng/mL; 210-TA, R&D Systems, Minneapolis, MN, USA) for 24 h at 37 °C. After incubation, cells were harvested for RNA isolation and conditioned media were collected for measuring secreted GM-CSF. Regarding the lipid metabolic signaling pathway inhibition study, MDA-MB-231 cells were preincubated with different inhibitors (myriocin (1 μM/mL; M1177-5MG), which is a serine palmitoyltransferase inhibitor (SPT-1) involved in sphingolipid biosynthesis; etomoxir (10 μM/mL; 236020), which is a carnitine palmitoyltransferase 1 (CPT-1), a mitochondrial enzyme involved in fatty acid β-oxidation; and triacsin C (5 μM/mL; T4540), which is a long-chain acyl-CoA synthetase 1 (ACSL1) inhibitor) and then treated with tumour necrosis factor-α (TNFα), (2 ng/mL; 210-TA, R&D Systems, Minneapolis, MN, USA). All lipid inhibitors were purchased from Sigma-Aldrich (Merck KGaA, Darmstadt, Germany). 

Regarding MAPK/TNFα signaling, cells were preincubated with different cell signaling pathway inhibitors for 1 h under cell culture conditions (SB203580 (10 µM/mL; 559389, Sigma-Aldrich, Merck KGaA, Darmstadt, Germany), a p38 inhibitor; PD98059 (10 µM/mL; tlrl-pd98, InvivoGen, San Diego, CA, USA), an inhibitor for ERK1/2; resveratrol (5 µM/mL; tlrl-resv, InvivoGen, San Diego, CA, USA), an inhibitor for NF-κB; and SP600125 (10 µM/mL; 420119, Sigma-Aldrich, Merck KGaA, Darmstadt, Germany), an inhibitor for JNK signaling pathway and then cells were stimulated for 8 h.

### 4.3. Real-Time Quantitative PCR

Total RNA was extracted from MDA-MB-231 cells using RNeasy Mini Kit (Qiagen, Valencia, CA, USA) as per the manufacturer’s instructions. The cDNA was synthesized using 1 μg of total RNA using a high-capacity cDNA reverse transcription kit (Applied Biosystems, Foster City, CA, USA). Real-time PCR was performed on a 7500 Fast Real-Time PCR System (Applied Biosystems, Foster City, CA, USA) using a TaqMan^TM^ Gene Expression Master Mix (Applied Biosystems, Foster City, CA, USA). Each reaction contained 500ng cDNA that was amplified with Inventoried TaqMan Gene Expression Assay products (CSF2, Hs00929873; ACSL1, Hs00960561; GAPDH, Hs03929097_g1). The threshold cycle (Ct) values were normalized to the house-keeping gene GAPDH and the amounts of target mRNA relative to control were calculated using the ΔΔCt-method [35,36]. Relative mRNA expression was expressed as fold expression over average of control gene expression. The expression level in the control treatment was assumed to be 1. Values are presented as mean ± SEM. Results were analyzed statistically; *p* < 0.05 was considered significant.

### 4.4. Small Interfering RNA (siRNA) Transfections

MDA-MB-231 cells were washed and re-suspended in 100 μL of nucleofector solution provided with the Amaxa Nucleofector Kit V and transfected separately with siRNA-ACSL1 (30 nM; OriGene Technologies, Inc., Rockville, MD, USA), scramble (control) siRNA (30 nM; OriGene Technologies, Inc., Rockville, MD, USA), and pmaxGFP (0.5 μg; Amaxa Nucleofector Kit V for MDA-MB-231 cells, Lonza, Basel, Switzerland). All transfection experiments were performed with an Amaxa Cell Line Nucleofector Kit V for MDA cells (Lonza, Basel, Switzerland) using an Amaxa Electroporation System (Lonza, Basel, Switzerland) according to the manufacturer’s protocol [37]. After 36 h of transfection, cells were treated with TNFα for 24 h. Cells were harvested for RNA isolation and staining study. ACSL1 gene knockdown level was assessed by real-time PCR using ACSL1 gene specific primer probes. 

### 4.5. GM-CSF Determination

Secreted GM-CSF protein in supernatants of MDA-MB-231 cells stimulated with TNFα was quantified using sandwich ELISA following the manufacturer’s instructions (DY215-05, R&D Systems, Minneapolis, MN, USA).

### 4.6. Western Blotting

MDA-MB-231 cells treated with TNFα at different time points were harvested and incubated for 30 min with lysis buffer (10X Lysis Buffer, Cell Signaling, Danvers, MA, USA). The protein lysates were prepared and resolved by 12% SDS-PAGE as described earlier [38]. Cellular proteins were transferred to an Immuno-Blot Polyvinylidene difluoride (PVDF) membrane (Bio-Rad Laboratories, Hercules, CA, USA) by electroblotting. The membranes were then blocked with 5% non-fat milk in PBS for 1 h, followed by incubation with primary antibodies against p44/42 MAPK (ERK1/2; cat# 9101) and 44/42 MAPK (ERK1/2; cat# 9102), p-p38 (cat# 9211) and p38 (cat# 9212), p-NF-κB (cat# 3033) and NF-κB (cat# 3034), and β-actin (cat# 4967) in 1:1000 dilution at 4 °C overnight. All primary antibodies were purchased from Cell Signaling (Cell Signaling Technology Inc., Danvers, MA, USA). The blots were then washed three times with TBS-T and incubated for 2 h with HRP-conjugated secondary antibody (Promega, Madison, WI, USA). Immunoreactive bands were developed using an Amersham ECL Plus Western Blotting Detection System (GE Healthcare, Chicago, IL, USA) and visualized by Molecular Imager® VersaDoc^TM^ MP Imaging Systems (Bio-Rad Laboratories, Hercules, CA, USA).

### 4.7. Immunocytofluorescence

MDA-MB-231 cells (10^6^/mL) were seeded on coverslips and cultured in 6-well plates at 37 °C. After incubation, the slides were then fixed in 4% formaldehyde and washed three times in cold PBS. Cells were then permeabilized in 0.1% Triton X-100, followed by three washes in cold PBS. The cells were blocked in 1% bovine serum albumin for 1 h. The slides were incubated overnight at room temperature with primary antibody of rabbit polyclonal anti GM-CSF antibody (GTX51383 Genetex, Irvine, CA, USA) in 1:200 dilution. The cells were then washed in PBS containing 0.05% Tween three times and again incubated with the secondary antibody conjugated with Alexa Fluor 694 or Alexa Fluor 488 (abcam® ab150079, abcam® ab150077) in 1:200 dilution for 1 h. After washing the slide several times in PBS, the cells were counterstained and mounted with coverslip using mountant containing DAPI (Vectashield H1500, Vectorlab, Burlingame, CA, USA).

The confocal images of the MDA-MB-231 cells were collected on an inverted Zeiss LSM710 AxioObserver microscope (Carl Zeiss, Gottingen, Germany) using a Plan-Apochromat 40×/1.40 oil DIC M27 objective lens. Excitation was via a 647 nm HeNe solid-state laser and the 405 nm line of an argon ion laser. After laser excitation of the samples, optimized emission detection bandwidths were configured using Zeiss Zen 2012 control software (version 1.1.1.2.0, Gottingen, Germany). Subsequently, the confocal images were captured, and fluorescence was measured using Zeiss Zen 2012 software (Gottingen, Germany).

### 4.8. Statistical Analysis

Statistical analysis was performed using GraphPad Prism software (version 6.07, La Jolla, CA, USA). Data are shown as mean ± standard error of the mean (SEM), unless otherwise indicated. One-way ANOVA followed by Tukey’s test were used to compare means between groups. For all analyses, data from a minimum of three sample sets were used for statistical calculation. *p*-value < 0.05 was considered significant. ns, no significance, *p < 0.05, **p < 0.01, ***p < 0.001, and ****p < 0.0001)
